# Lipid Droplet Binding of Hepatitis C Virus Core Protein Genotype 3

**DOI:** 10.5402/2012/176728

**Published:** 2012-07-11

**Authors:** Guan Qiang, Ravi Jhaveri

**Affiliations:** ^1^Division of Infectious Diseases, Department of Pediatrics, Duke University Medical Center, Durham, NC 27710, USA; ^2^Department of Molecular Genetics and Microbiology, Duke University School of Medicine, Durham, NC 27710, USA

## Abstract

*Background*. Hepatitis C virus (HCV) genotype 3 is known to cause steatosis (fatty liver) that is more frequent and severe than other genotypes. We previously identified sequence elements within genotype 3 HCV Core domain 3 that were sufficient for lipid accumulation. *Aims*. We examined various genotype 3 Core domains for lipid droplet localization and compared the lipid droplet binding regions of domain 2 with a genotype 1 isolate. *Methods*. We generated HCV Core domain constructs fused with green fluorescent protein and performed immunofluorescence to visualize lipid droplets. *Results*. Constructs containing HCV Core domain 2 are appropriately localized to lipid droplets with varying degrees of efficiency. When compared to genotype 1, there are polymorphisms within domain 2 that do not appear to alter lipid droplet localization. *Conclusions*. In summary, the differences in a steatosis-associated HCV Core genotype 3 isolate do not appear to involve altered lipid droplet localization.

## 1. Introduction

Hepatitis C virus (HCV) is a major cause of liver disease globally, a significant cause of chronic liver disease and the leading indication for adult liver transplantation in the United States [1–3]. HCV has been shown to manipulate host lipid metabolism on a number of different levels, from altered serum cholesterol levels at the patient level to significant rates of steatosis at the organ level finally to significant associations with lipid droplets in liver cells *in vitro  *[4–15]. Studies on the pathogenesis of HCV and alterations in lipid metabolism have frequently implicated the Core protein. Recent studies have shown that HCV uses the VLDL synthesis pathway for virus release, and Core protein binding to lipid droplets is required for efficient virus assembly, both are integral parts of the viral lifecycle [13, 14, 16]. This is consistent with prior observations that *in vitro* expression of Core protein from a variety of genotypes leads to lipid accumulation [17–19].

The Core (nucleocapsid) protein is composed of 3 domains based on hydrophobicity profiling and has been associated with altering a diverse range of intracellular pathways [20–25]. Domain 3 is the highly hydrophobic “signal peptide” region that facilitates 2 cleavage events: at the Core-E1 junction by signal peptidase and at the domain 2-3 junction by signal peptide peptidase [20, 25–27]. Proper cleavage of Core has been shown to be an essential step in the production of HCV viral particles [24, 28, 29].

Previous work on HCV Core protein and lipid droplet binding has focused on domain 2 of the protein [30]. The helix II portion of domain 2 was found to be sufficient to localize a GFP fusion construct to lipid droplets [31]. Amino acid residue F164 within domain 2 from genotype 3 was also determined to increase fatty acid synthase expression compared to the genotype 1 tyrosine residue [32, 33].

In previous work on examining viral factors associated with lipid accumulation in HCV genotype 3 infection, we identified specific polymorphisms at amino acid residues 182 and 186 within domain 3 of the Core protein that correlated with the presence or absence of steatosis in a small group of patients [18]. We have subsequently shown that domain 3 alone is sufficient for the accumulation of lipid, providing evidence that domain 3 plays some role beyond just being the “signal peptide” [34]. 

In this study we characterized the relationship between HCV Core domains 1, 2, and 3 from a steatosis-associated genotype 3 isolate in determining lipid droplet localization. We hypothesized that a steatosis-associated domain 3 may alter lipid droplet localization of this isolate. We created HCV Core deletion constructs fused to green fluorescent protein (GFP), performed transient transfections, and performed light and immunofluorescent microscopy to assess lipid droplet localization. We also performed a sequence comparison to a genotype 1 isolate, focusing on the regions responsible for lipid droplet localization. 

## 2. Material and Methods

### 2.1. HCV Constructs

 HCV1 DCRI (GenBank ID EU099414), a genotype 3 Core constructs we cloned from a patient sample was used as template for all subsequent constructs discussed. Sequence from the H77C isolate (GenBank ID AF011751.1), a genotype 1a, was also used for cloning and sequence comparison. 

The plasmid named “78,” a gift from Dr. Brian Doehle, which contained the backbone of pcDNA3 ligated with a fragment from pEGFP-C, containing part of the CMV promoter, the GFP ORF, and part of the multiple cloning site, after both were digested with *NdeI* and *EcoRI*.

Untagged domain 1-2 constructs were cloned into a pcDNA 3.1 V5-His A vector (Invitrogen, Carlsbad, CA, USA) using EcoR1 and Xba1 digestion.

### 2.2. Plasmid Construction

Plasmids encoding GFP fusion proteins were generated using PCR and gene-specific primers listed in Table 1 to generate HCV Core protein full-length and deletion mutants encoding domains 2 + 3, domains 1 + 2, and domains 2 and 3 individually from the HCV1 gene. Amplicons were digested with either BspEI or EcoRI and XbaI and subsequently cloned into digested and purified vector. For untagged domain 1-2 constructs, EcoR1 and Xba1 were used for digestion. Ligated products were transformed into competent *E. coli* and colonies were selected after overnight growth of Luria-Bertani agar containing Ampicillin. Recombinant plasmids were purified and sequenced to verify code and frame.

### 2.3. Immunoblotting

All cell lysates were prepared using Passive Lysis Buffer (Promega, Madison, WI, USA) and analyzed by immunoblot using the following primary antibodies: green fluorescent protein (Abcam, Cambridge, MA, USA), *β*-actin (GenScript, Piscataway, NJ, USA), and the following secondary antibodies: goat anti-mouse-HRP (GenScript, Piscataway, NJ).

### 2.4. Transient Transfections

Huh7.5 human hepatoma cells were passaged into 4-well chamber slides and were transfected the next day when cells were approximately 70% confluent. One microgram plasmid DNA was used for each well combined with JetPEI reagent (PolyPlus, Illkirch, France) as per the manufacturer's protocol. Cells were incubated for 24–48 hours and then fixed and stained with either protocol described below.

### 2.5. Immunofluorescence

A chicken polyclonal antibody to Adipose differentiation-related protein (ADRP) (Abcam) was used for immunofluorescence experiments. For untagged domain 1-2 constructs, an HCV Core monoclonal antibody (ThermoPierce, Rockford, IL, USA) was used for immunofluorescence. Staining was performed as described previously where primary antibody staining was performed for 30 minutes at 37°C, followed by 2 PBS washes, and then stained with a secondary rabbit anti-chicken antibody conjugated to Rhodamine (Abcam) for 30 minutes at 37°C [18]. After 2 PBS washes and a brief fixation step using 4% paraformaldehyde, staining procedure proceeded as described previously with DAPI stain without the Oil Red O component [18]. Slides were examined using Axiovert 200 microscope (Carl Zeiss) with epifluorescent illumination, and images were recorded using an AxioCam HRC camera (Carl Zeiss) and AxioVision 4.4 software (Carl Zeiss) using the same settings for all photographs.

## 3. Results

**Figure 1:**
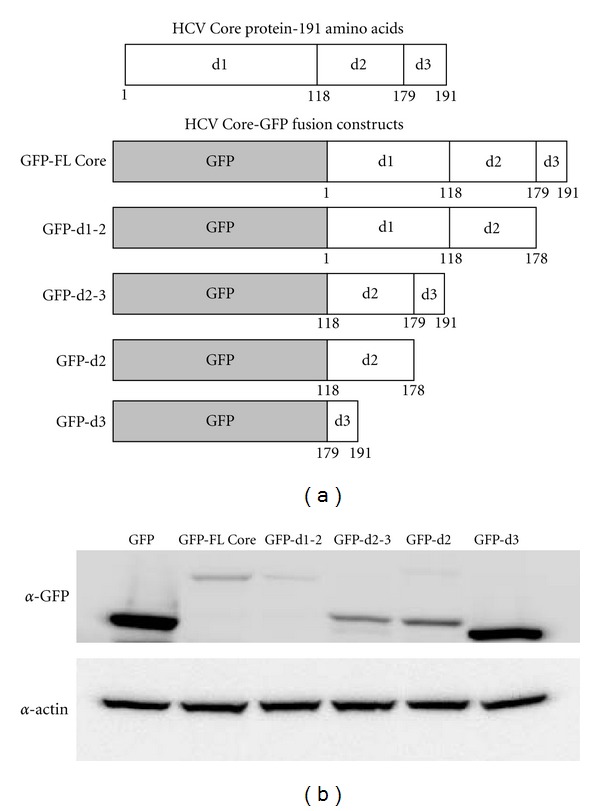
Overview of GFP fusion constructs. (a) GFP fusion constructs were generated with various full-length and deletion constructs of HCV Core protein. Each construct is presented with the amino acid residue boundaries included. GFP-FL Core: full-length Core protein, GFP-d1-2: Core domains 1 and 2, GFP-d2-3: Core domains 2 and 3, GFP-d2: Core domain 2 alone, and GFP-d3: Core domain 3 alone. (Construct labels used in the figure are consistent through all figures.) (b) Construct expression was validated using transient transfection of Huh7.5 cells and western blotting. Each construct produced a band at the predicted protein size.

We created GFP fusion constructs expressing full-length Core, domains 1-2 alone, domains 2-3 alone, domain 2 alone and domain 3 alone (Figure 1(a)) with the boundaries of each domain listed under each construct. Using transient transfection of Huh7.5 cells, we performed western blot analysis using a GFP antibody to confirm appropriate expression of our constructs. All constructs showed a band at the expected size and the relative size differences between the constructs were appropriate (Figure 1(b)). It should be noted that expression levels of full-length Core protein and domains 1-2 were lower than the other constructs. This instability has been independently described previously by several groups [21, 27, 35, 36].

We initially transfected each construct into Huh7.5 cells and analyzed cells expressing each using a combination of fluorescent microscopy and ORO to stain neutral lipid containing droplets (data not shown). All constructs expressed appropriately with one exception. Cells expressing domains 1-2 fused to GFP did not colocalize to lipid droplets but rather had an almost exclusive nuclear localization. We performed subsequent analysis by confirming the sequence of the original construct and removing the GFP tag. We demonstrated that this nuclear localization was an artifact of the construct. As would be expected from previously published data using domain 1-2 deletion constructs from a genotype 1 isolate, an untagged version is localized appropriately to lipid droplets, and this untagged construct was used for the rest of the experiments [30, 31].

**Figure 2:**
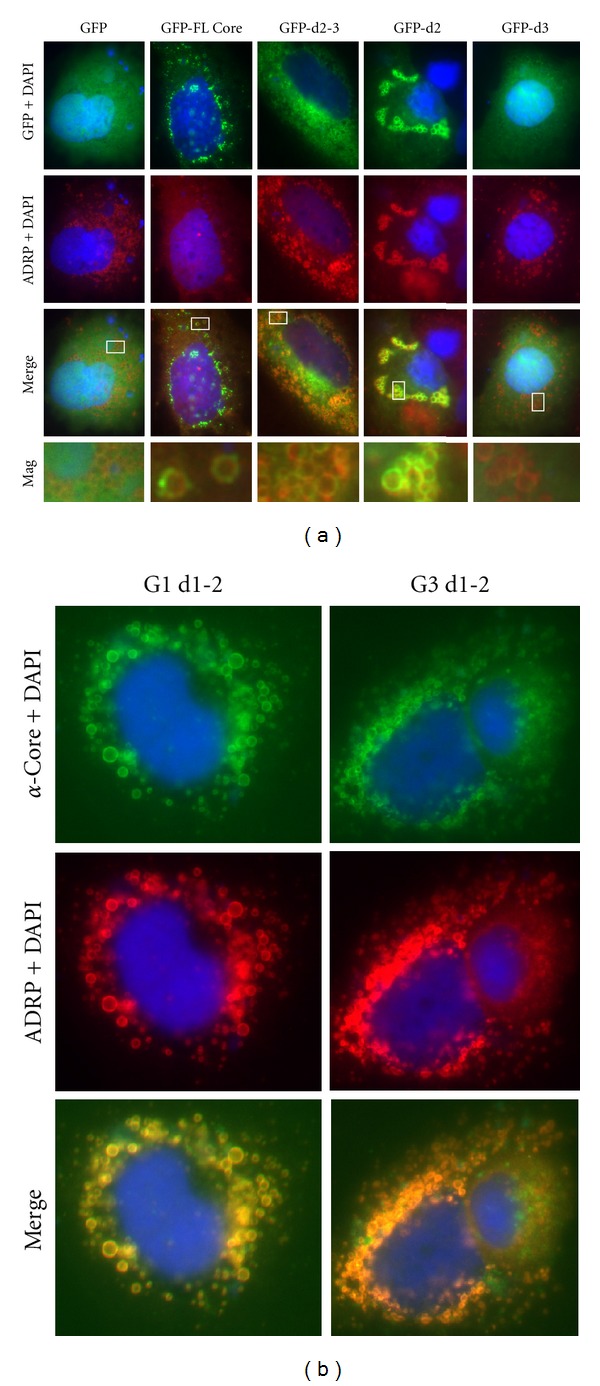
Colocalization of GFP-Core fusion constructs with ADRP in transfected cells. (a) Cells expressing GFP-Core fusion constructs were analyzed using immunofluorescence with antibodies to ADRP. Separate green channel (GFP) and red channel (ADRP) images are presented as well as a merged image (Merge) to illustrate colocalization as defined by yellow overlap of the green and red fluorescence. A portion of each merged image (white box) was magnified (Mag) to provide a more detailed view of fluorescent overlap. DAPI was used as a nuclear counterstain. (b) Cells expressing untagged HCV Core domain 1-2 construct from genotype 1 (G1 d1-2) and steatosis-associated genotype 3 (G3 d1-2). Cells were analyzed using HCV Core monoclonal antibody (*α*-Core, green channel), ADRP antibody (red channel) and a merged image (yellow overlap). DAPI was used as a nuclear counterstain.

Because ORO only stains neutral lipids, is not a specific marker for lipid droplets, and may alter the appearance of lipid droplets, we used an antibody to ADRP to define lipid droplet localization [37]. Cells expressing GFP showed broad cellular fluorescence with no overlap with ADRP fluorescence (Figure 2(a)). Cells expressing full-length Core, domain 2-3 together or domain 2 demonstrated colocalized with lipid droplets as indicated by significant yellow overlap of the green-red fluorescent signal in the merged images. Close examination of these images demonstrates “rings” of green fluorescence around lipid droplets. Cells expressing the domain 3 alone construct did not demonstrate any overlap of green-red fluorescent signal. Localization of domain 3 from the genotype 1 isolate away from the lipid droplet was identical to genotype 3 (data not shown). Examination of untagged domain 1-2 constructs from both the steatosis-associated genotype 3 and the H77C genotype 1a showed that each localized to lipid droplets with almost complete overlap with ADRP (Figure 2(b)). The genotype 3 isolate did not appear to alter the morphology of lipid droplets compared to genotype 1a.

**Figure 3:**
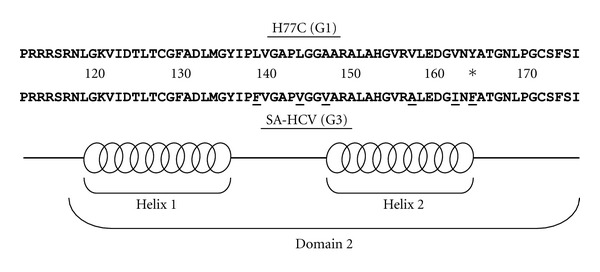
Sequence comparison of HCV Core domain 2 from genotype 1 and genotype 3 isolates. Genotype 1 H77C (H77C-G1) and steatosis-associated genotype 3 (SA-HCV G3) were compared from amino acid positions 112–176 for sequence differences within domain 2. Sequence differences are underlined and the span of and helical regions within domain 2 based on the prior literature are indicated by the bracket and clustered ovals in the diagram [31].

We performed sequence comparison of the genotype 3 and genotype 1 isolates, focusing on regions within domain 2 that have been previously identified to be necessary for lipid droplet localization (Figure 3) [30, 31]. We found several synonymous and nonsynonymous polymorphisms in the genotype 3 isolate (L139F, L144V, A147V, V157A, V162I, and Y164F). These were focused in the interhelical region and in helix 2 of domain 2, including the phenylalanine at position 164, which is unique to genotype 3 isolates. There were no polymorphisms detected in helix 1.

## 4. Discussion

In this study, we sought to further define the differences with HCV Core genotype 3 that contribute to clinical steatosis and *in vitro* lipid accumulation. The significant differences with genotype 3 core protein that have been highlighted are the phenylalanine at position 164 and sequence polymorphisms within domain 3. Our results here demonstrate that neither of these differences contribute to significant differences in lipid droplet localization between genotype 3 and genotype 1 isolates. Deletion constructs from genotype 3 Core required domain 2 to localize to lipid droplets and sequence differences between genotype 3 and genotype 1 isolates within domain 2 did not occur within the critical regions for lipid droplet localization.

These results further separate the phenomena of lipid droplet localization and intracellular lipid accumulation when examining the differences in HCV genotype 3 isolates. Previous studies have focused on regions within domain 2 being important for lipid droplet localization [31–33]. In a previous study, we hypothesized that based on our findings in domain 3 that these 2 phenomena may be mediated by 2 distinct regions of the Core protein [18]. We have recently published data demonstrating that domain 3 alone is sufficient for lipid accumulation [34]. These results demonstrate that lipid droplet localization is independent of lipid accumulation and not significantly different between genotypes.

In the initial work describing regions within domain 2 that were required for lipid droplet binding, Hope and McLauchlan used deletion mutants of domain 2 and Boulant et al. constructed GFP fusion constructs [30, 31]. Our findings with the genotype 3 domain 2 alone construct are consistent with their observations. Okamoto et al. demonstrated previously the role of signal peptide peptidase (SPP) cleavage of Core in the localization to lipid droplets, while Targett-Adams et al. showed that inhibition of SPP decreased titers of virus in the JFH HCV cell culture virus system [24, 29]. Pène et al. reported that SP cleavage must occur prior to SPP cleavage of Core protein [28]. Our constructs were not designed to assess differences in SPP cleavage between genotype 3 and genotype 1 constructs, but we do not presume that there are significant differences based on sequence conservation at and around the SPP cleavage site.

When analyzing the sequence differences in domain 2 between genotype 3 and genotype 1 isolates, several things are notable. One is that all of the sequence differences are within the interhelical region or helix 2. Based on the work of Boulant et al., these 2 regions were absolutely essential for association with lipid droplets [31]. However, when looking at the NMR data from this paper, the changes in the genotype 3 isolate do not involve the charged residues within these regions, and thus do not disrupt the amphipathic characteristics of the helix. The differences in genotype 3 (Phe139, Val144, Val147, Ala157, and Ile162) all preserve the helical structure in this region and specific residues 139, 144, and 147, that Boulant et al. proved in genotype 1 were critical for lipid droplet localization [31]. It is also interesting to note that Phe164, which is a sequence difference that is unique to genotype 3 and has been identified as one of the major determinants for lipid accumulation due to its effect of Fatty acid synthase, plays no role in lipid droplet localization [32, 33].

Our results should be analyzed in the light of certain limitations. First, we used GFP fusion constructs which raises concern about the context of these results. However, our full-length GFP fusion and our domain 2 alone fusion both localized to lipid droplets, which is as predicted based on previously published work. The second limitation is this system lacks the context of other viral proteins and the entire viral life cycle. Again, these results are consistent with previous results using the JFH cell culture system.

In conclusion, we have demonstrated that HCV Core genotype 3 exhibits the same requirements for lipid droplet localization as genotype 1 isolates and that sequence differences observed in genotype 3 do not alter the critical residues involved in this process. These results further separate lipid droplet localization from intracellular lipid accumulation in the mechanisms of steatosis formation in HCV genotype 3 infections.

## Figures and Tables

**Table 1:** Primer sequences in plasmid construction.

GFP fusion constructs	
FL Core EcoR1 sense	
GTG CGA ATT CGA TGA GCA CAC TTC CTA AA	
d2 EcoR1 sense	
GTG CGA ATT CGA ACT TGG GTA AAG TCA TCG	
FL core Xba1 antisense	
AGT CTC TAG ATC ATC AAC TTG CTG CTG GAT G	
d2 Xba1 antisense	
ATG CTC TAG ATC ATC AAA GGA AGA TAG AAA AGG AGC AAC CG	
d3 EcoR1-Xba1 sense	
AAT TCC CTT GCT TTG TTC TCT TGC TTA GTT CAT CCA GCA GCA AGT TGA TGA T	
d3 EcoR1-Xba1 antisense	
CTA GAT CAT CAA CTT GCT GCT GGA TGA ACT AAG CAA GAG AAC AAA GCA AGG G	

Untagged HCV Core constructs	

HCV Core genotype 3 sense	
ATG CGA ATT CGC CAC CAT GAG CAC ACT TCC TAA A	
HCV Core genotype 1 sense	
ATG CGA ATT CGC CAC CAT GAG CAC GAA TCC TAA A	
HCV Core genotype 1 d2 Xba1 antisense	
ATG CTC TAG ATC ATC AAA GGA AGA TAG AGA AAG AGC AAC CA	
